# The miR‐146a/*IGSF11* Axis Potentially Mediates the Protective Effect of Dexmedetomidine Against Cigarette Smoke–Induced Chronic Obstructive Pulmonary Disease

**DOI:** 10.1155/humu/1858420

**Published:** 2026-05-13

**Authors:** Xiao Chen, Dan Wang, Jing Du, Jiale Li, Zihe Wang, Binbin Chen, Xueqing Yao, Shuangfeng Li, Na Li

**Affiliations:** ^1^ Department of Anesthesiology, Hainan Affiliated Hospital of Hainan Medical University (Hainan General Hospital), Haikou, China, hainmc.edu.cn; ^2^ Department of Anesthesiology, Hainan General Hospital (Hainan Affiliated Hospital of Hainan Medical University), Haikou, China, hainmc.edu.cn; ^3^ Medical Laboratory Center, Hainan General Hospital (Hainan Affiliated Hospital of Hainan Medical University), Haikou, China, hainmc.edu.cn; ^4^ Department of Anesthesiology, Haikou Maternal and Child Health Hospital, Haikou, China

**Keywords:** chronic obstructive pulmonary disease, cigarette smoke extract, dexmedetomidine, immunoglobulin superfamily Member 11, microRNA-146a

## Abstract

**Background:**

This study comprehensively examined the mechanism of the roles of dexmedetomidine (Dex) and miR‐146a in chronic obstructive pulmonary disease (COPD).

**Methods:**

COPD models were established employing cigarette smoke extract (CSE)–exposed airway epithelium cell 16HBE and cigarette smoke–treated Sprague–Dawley rats, followed by interference with Dex and transfection. CCK‐8 and flow cytometry assays were employed for the detection of cell viability and apoptosis. Further, the levels of factors related to the NF‐*κ*B pathway, inflammation, and apoptosis were measured by ELISA, qRT‐PCR, and Western blot.

**Results:**

Dex and miR‐146a mimic notably inhibited the inflammation and apoptosis of CSE‐induced 16HBE cells via modulating the levels of TNF‐*α*, IL‐6, IL‐8 (inflammatory cytokines), and caspase‐3, Bcl‐2, and Bax (apoptosis‐associated proteins), whereas miR‐146a inhibitor exerted opposite effects. Moreover, Dex modulated the levels of both miR‐146a and its downstream target immunoglobulin superfamily Member 11 (*IGSF11*). The protective effects of miR‐146a on COPD were reversed following *IGSF11* intervention. Further, the phosphorylation of NF‐*κ*B and IKK*γ* suggested that the effects of Dex/miR‐146a/*IGSF11* axis on COPD were related to the NF‐*κ*B pathway.

**Conclusion:**

Our in vitro and in vivo studies showed that Dex alleviated CSE‐induced COPD via modulating the miR‐146a/*IGSF11*/NF‐*κ*B axis.

## 1. Introduction

Exposure to inhaled noxious particles, tobacco smoke, and pollutants will lead to the development of chronic obstructive pulmonary disease (COPD) [1, 2], an obstructive disease with high morbidity and mortality [3]. The global incidence of COPD among individuals aged 30–79 years ranges from 7.6% to 10.6%, with the highest burden in low‐ and middle‐income countries. Additionally, the associated risks of comorbidities and sudden death in this population [4] underscore the urgent need to identify COPD‐related therapeutic targets and to elucidate their underlying pathways.

Studies show that *α*_2_‐adrenergic receptor agonists can cause histopathological changes in sheep lung tissue [5]. Dexmedetomidine (Dex) can exert antioxidative stress, antiapoptotic, and anti‐inflammatory effects on diverse organs [6]. Owing to these pleiotropic properties, growing evidence demonstrates its protective effects on the heart, liver, and spleen as well as on lung function [7]. For instance, Dex exhibits anti‐inflammatory efficacy in LPS‐induced acute lung injury (ALI) by modulating HMGB1‐mediated TLR4/NF‐*κ*B pathway [8]. Also, Dex attenuates ALI‐modeled rats by promoting the differentiation of Tregs via activating AMPK/SIRT1 pathway [9]. Dex also contributes to the treatment of COPD through downregulating PACER [10], for instance, it ameliorates oxidative stress responses and alleviates lung tissue inflammation in COPD rats during mechanical ventilation [11].

Dex has been found to regulate apoptosis in human bronchial epithelial cells mimicking COPD via miR‐146a [12]. miR‐146a belongs to the family of microRNAs (miRNAs), which are highly conserved small noncoding RNAs with ~22 nucleotides in length. These molecules participate in diverse biological pathways by binding to target messenger RNAs (mRNAs), leading to mRNA degradation or translational inhibition [13]. For example, low‐expressed miR‐195‐5p can reduce the generation of proinflammatory cytokines in pulmonary macrophages and attenuate pulmonary inflammatory response in COPD rats [14, 15]. Also, suppression of miR‐24‐3p enhances cigarette smoke (CS)–induced emphysema and epithelial apoptosis in mice [16], and miR‐377‐3p exacerbates COPD by driving bleomycin‐induced senescence of lung fibroblasts [17]. miR‐146a is recognized as a critical regulator of innate and adaptive immune responses and is implicated in the pathogenesis of some chronic inflammatory diseases [18]. In addition, miR‐146a methylation affects lung function in patients with COPD [19], and miR‐146a can prevent the formation of emphysema and related abnormal inflammatory responses in mice [20]. Adenosine methylation of miRNAs such as miR‐125a‐5p could regulate the expression of *IGSF11* in lung cancer cells to promote immune escape [21]. *IGSF11* is high‐expressed in tumors and is regulated by various factors [22]. A previous study indicates that *IGSF11* can promote the migration and invasion of melanoma cells and enrich the gene set associated with epithelial–mesenchymal transition (EMT) [23]. However, the relationship between *IGSF11* and miR‐146a remains unclear, and it was unknown whether Dex exerted a synergistic effect on COPD through the miR‐146a/*IGSF11* axis. Hence, the present work investigated the role of Dex, miR‐146a, and *IGSF11* in COPD using a CSE‐exposed airway epithelial cell model and a CS‐exposed rat model.

## 2. Material and Methods

### 2.1. CSE Preparation

CSE was prepared following a previously established method with minor modifications [24]. Nonfilter cigarettes (brand: Daqianmen, tar: 13 mg, nicotine: 1.3 mg, Shanghai Tobacco Company, Shanghai, China) were combusted using a peristaltic pump and bubbled through 10 mL of serum‐free F‐12K medium in a collector at a constant flow rate of 1 L/min until the cigarette was consumed to 0.5 cm from the end. The collector was gently shaken for 10 min until there was no visible smoke. Then, the solution was transferred to another container. The pH was adjusted to 7.2–7.4 with 1‐mM sodium bicarbonate, followed by sterile filtration through a 0.22‐*μ*m filter. The absorbance at the wavelength of 320 nm was measured for standardization and stored in a refrigerator at 4°C. The prepared solution was considered as 100% CSE and diluted to 2%, 5%, 10%, 20%, and 50% within 1 h of use.

### 2.2. Cell Culture and Intervention

DMEM (Cellgro, Manassas, Virginia) added with 1% penicillin–streptomycin–amphotericin B solution (Cellgro) and 10% FBS (Cellgro) was used to culture human airway epithelial cells 16HBE (Millipore, Darmstadt, Germany, RRID: CVCL_0112) in an incubator at 37°C with 5% CO_2_. The authenticated cell line was free of mycoplasma contamination.

Then, 16HBE cells were exposed to specified concentrations of CSE and/or Dex at 1, 5, 10, and 15 *μ*M for 24 h as needed. miR‐146a mimic/inhibitor and their controls and overexpression plasmid of *IGSF11* (hereafter oe‐*IGSF11*) were obtained from RiboBio (Guangzhou, China). The transfection was conducted applying Lipofectamine 2000 transfection reagent (Invitrogen, Carlsbad, California) following the protocols.

### 2.3. CCK‐8 Assay

The viability of 16HBE cells following various interventions was measured using a CCK‐8 assay kit following the manuals. In detail, 16HBE cells were planted at 1 × 10^5^ cells/mL into 96‐well plates and cultured for 12, 24, 36 and 48 h. Subsequently, 10‐*μ*L CCK‐8 reagent was added to each well for 4‐h incubation. Finally, the absorbance at 450 nm was read by a microplate reader (MCC/340, ThermoFisher Scientific, Hudson, New Hampshire) [25].

### 2.4. Flow Cytometry

Following the interventions, the working solution of Annexin V‐FITC and PI (Miltenyi Biotech, Bergisch Gladbach, Germany) along with the binding solution (200 *μ*L) were added to the cells (1 × 10^5^ cells in total). After rinsing the cells in PBS, cell apoptosis was determined applying Accuri C6 flow cytometer (BD Biosciences, San Jose, California) [26].

### 2.5. Prediction of the Binding Between miRNA and the 3 ^′^ UTR of *IGSF11*

The mature sequence of miR‐146a‐3p (5 ^′^‐CCUCUGAAAUUCAGUUCUUCAG‐3 ^′^) was investigated for its interaction with *IGSF11*. Given that miRNAs typically exert degradation effect through binding to the 3 ^′^ UTR of target genes, we identified the corresponding 3 ^′^ UTR sequence of *IGSF11* (Table S1). The sequences were submitted to the AlphaFold Server (https://alphafoldserver.com) for molecular structure fusion prediction. The “Illustrative” option in Quick Styles was selected for 3D modeling to locate the spatial position of the mature miR‐146a‐3p and to identify the matching 3 ^′^ UTR sequence of *IGSF11*, which was recognized as the potential binding site of miR‐146a‐3p.

### 2.6. Dual‐Luciferase Reporter Assay

RPMI‐1640 medium with 10% heat‐inactivated FBS and 1% penicillin–streptomycin was used to culture human 293 cell line (ATCC, Manassas, Virginia, RRID: CVCL_0045) at 37°C in an environment with 5% CO_2_.

The sequence fragment of *IGSF11* containing the putative or abrogated binding sites of miR‐146a was inserted into the pmiRGLO vector (Promega, Madison, Wisconsin) to construct the luciferase reporter plasmids *IGSF11*‐WT and *IGSF11*‐MUT. These plasmids were then cotransfected with miR‐146a mimic/mimic‐NC into 293 cells following the manuals. The luciferase activity at 48 h was finally read in the dual‐luciferase reporter system (Promega) [27].

### 2.7. Rat COPD Model

Our study has been carefully reviewed and approved by the Ethics Committee. Animal experiments were carried out strictly following the guidelines of the China National Council on Laboratory Animal Welfare and possible efforts were devoted to minimize suffering to the animals.

SD rats (body weight: 230 ± 25 g, age: 8 weeks, Shanghai Laboratory Animal Center, Shanghai, China) were reared in the facility at room temperature of 26°C under a 12‐h light–dark cycle. Free access to diet and water was provided.

A CS‐induced rat COPD model was developed as previously described, with minor modifications [28]. In detail, modeled rats (*n* = 3) were exposed to four concurrently burning cigarettes three times per day (1 h per time), 5 days a week for 16 weeks using a custom‐designed nose‐only directed flow inhalation and smoke exposure system. Rats in the control group were exposed to normal air under the same schedule.

Following model establishment, Dex was administered via intravenous drip at 1 *μ*g/kg/h [11]. For miR‐146a overexpression, an adenovirus carrying miR‐146a mimic was injected into rats at 1 × 10^8^ viral particles [14].

Rats were anesthetized via 5% isoflurane inhalation and sacrificed. Blood samples were collected via carotid artery puncture and further centrifuged (12,000 × g at 4°C for 15 min) to obtain serum. Meanwhile, the chest was opened to obtain lung samples, which were stored at −80°C for subsequent assays.

### 2.8. ELISA

Using a commercial assay kit purchased from Lianke Bio [29], the concentration of inflammatory cytokines (TNF‐*α*, IL‐8, and IL‐6) in the culture supernatant of 16HBE cells and rat serum samples was measured according to the instructions.

### 2.9. Quantitative Real‐Time PCR

Total RNA from lung tissues and 16HBE cells was extracted by TriZol (Invitrogen) and then reverse‐transcribed into the cDNA using PrimeScript Reagent Kit (ThermoFisher Scientific). The PCR was then initiated in ABI‐PRISM 7500 Real‐Time PCR System (Applied Biosystems, Carlsbad, Californa) using SYBR Premix Ex Taq II (Takara, Shiga, Japan). Using the formula 2^-*ΔΔ*Ct^_,_ relative gene expressions from independent triplicate tests were calculated, with *GAPDH* and *U6* as housekeeping controls [30]. The primers used were displayed in Table 1.

**Table 1:** Sequences of primers.

Gene	Forward primer sequence (5 ^′^‐3 ^′^)	Reverse primer sequence (5 ^′^‐3 ^′^)
IGSF11 (human)	TTACCTTTGGGAGAAGTTAG	ACCAGAGTCTTATGTTGACC
hsa‐miR‐146a	GGGTTGTCGTATCCAGTGCAA	GTCGTATCCAGTGCGTGTCG
Igsf11 (rat)	TTACCTTTGGGAGAAGTTAG	CAGTGTAGGAATGTGTGTGT
rno‐miR‐146a	CCATGGGTTGTCGTATCCAGT	GTATCCAGTGCGTGTCGTGG
Caspase‐3 (rat)	CATGGAGATGAAGGAGTAAT	TTCCACTGTCTGTCTCAATA
Bax (rat)	TGAACAGATCATGAAGACAG	TGCTAGCAAAGTAGAAAAGG
IKK*γ* (rat)	AATCAGTTGAGTGAGATGGT	CTCTAAAGTTTGTCGTTCCT
NF‐*κ*B (rat)	AGAGAAGCACAGATACCACT	GTTATTGTTGGTCTGGATG
GAPDH (human)	AGGTCGGTGTGAACGGATTTG	TGTAGACCATGTAGTTGAGGTCA
Gapdh (rat)	TGAGTATGTCGTGGAGTCTA	CACAAAGTTGTCATTGAGAG
U6 (human)	ACAGAGAAGATTAGCATGGCC	GACCAATTCTCGATTTGTGCG
U6 (rat)	TACAGAGAAGATTAGCATGGCCC	ACGAATTTGCGTGTCATCCT

### 2.10. Western Blot

Total protein sample was separated from lung tissue homogenate and 16HBE cells using the ice‐cold RIPA lysis buffer containing proteinase and phosphatase inhibitors. Protein concentration was measured with BCA protein assay kit (Solarbio, Beijing, China). Then, 30‐*μ*g protein sample was separated via electrophoresis and moved onto the PVDF films, which were blocked via 5% defatted milk and incubated with the primary antibodies against caspase‐3 (1:1000, Novus Biological, Centennial, Colorado), Bax (1:1000, CST, Danvers, Massachusetts), Bcl‐2 (1:1000, ABclonal, Wuhan, China), IGSF‐11 (1:1000, Abcam, Cambridge, United Kingdom), NF‐*κ*B (1:1000, Novus Biological), p‐NF‐*κ*B (1:1000, Novus Biological), I*κ*B kinase gamma (IKK*γ*, 1:1000, Novus Biological), p‐IKK*γ* (1:2000, ABclonal), and GAPDH (1:3000, ZSGB, Beijing, China) at 4°C overnight. After washing three times, goat antimouse IgG antibodies or horseradish peroxidase–conjugated goat antirabbit IgG (1:5000, ZSGB) were used to incubate with the film at room temperature for 2 h. The bands were developed applying ECL solution (Merck KGaA, Darmstadt, Germany) and data visualization was achieved using the ChemiDoc touch imaging system (Bio‐Rad, Hercules, California). The grey scale of the protein bands was quantified via ImageJ.

### 2.11. Data Analysis

Data from three replicates were analyzed using GraphPad Prism 8.0 software (GraphPad Prism, San Diego, California) and expressed as mean ± SD. In the exposure and treatment procedures, all experimental groups were assigned using a randomization method. To minimize subjective bias, outcome assessors were blinded to the experimental group assignments throughout the experimental process. Statistical analyses were conducted with one‐way ANOVA, followed by Tukey′s post hoc test. *p* < 0.05 was used to define statistical significance.

## 3. Results

### 3.1. Effects of Different Concentrations of CSE on the Inflammatory Cytokines in 16HBE Cells

**Figure 1:**
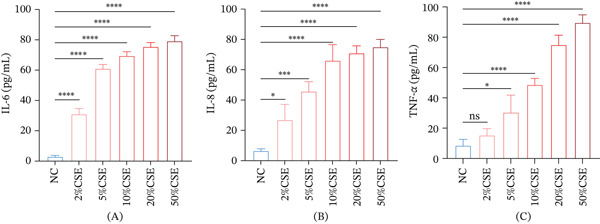
Effects of CSE at different concentrations on the inflammatory cytokines in 16HBE cells. (A–C) Results of ELISA assay on the concentrations of (A) IL‐6, (B) IL‐8, and (C) TNF‐*α* in 16HBE cells with diverse interventions. ns: nonsignificant;  ^∗^ *p* < 0.05,  ^∗∗∗^ *p* < 0.001,  ^∗∗∗∗^ *p* < 0.0001.

A COPD cell model was established by exposing 16HBE cells to CS for 24 h, and we measured the concentration of inflammatory cytokines IL‐6 (Figure 1A), IL‐8 (Figure 1B), and TNF‐*α* (Figure 1C) in 16HBE cells. ELISA assay showed that CSE at diverse concentrations all increased the levels of these cytokines in 16HBE cells (Figure 1A–C, *p* < 0.05).

### 3.2. Protective Effects of Dex on CSE‐Induced Inflammation and Apoptosis of 16HBE Cells

**Figure 2:**
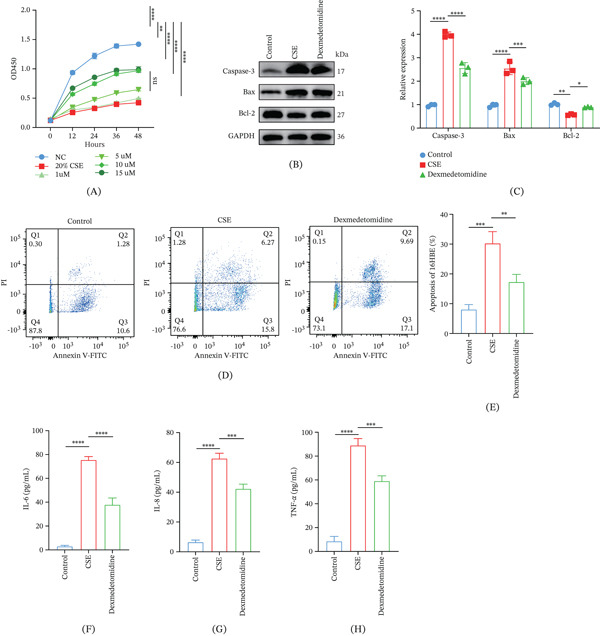
Protective effects of Dex on CSE‐induced apoptosis and inflammation of 16HBE cells. (A) CCK‐8 assay results showing the optimal concentration of Dex. (B, C) Relative expression level of Bcl‐2, caspase‐3, and Bax (three apoptosis‐related proteins) in 16HBE cells. (D, E) Flow cytometry measured apoptosis (%) of 16HBE cells. (F–H) Results of ELISA assay on the concentrations of (F) IL‐6, (G) IL‐8, and (H) TNF‐*α* in 16HBE cells with diverse interventions. ns: nonsignificant,  ^∗^ *p* < 0.05, ^∗∗^ *p* < 0.01, ^∗∗∗^ *p* < 0.001, ^∗∗∗∗^ *p* < 0.0001.

CCK‐8 assay showed that in COPD model cells, all concentrations of Dex (1, 5, 10, and 15 *μ*M) evidently promoted the viability of CSE‐treated cells, as evidenced by the increased OD450 value in these cells (Figure 2A, *p* < 0.01). Since 10‐ and 15‐*μ*M Dex demonstrated comparable efficacy in significantly enhancing the viability of CSE‐induced 16HBE cells, the lower and more cost‐effective concentration (10 *μ*M) was selected for further experiments.

Following CSE exposure and Dex treatment, it was observed that CSE promoted the apoptosis of 16HBE cells, as evidenced by decreased level of Bcl‐2 and increased levels of caspase‐3 and Bax (Figure 2B–E, *p* < 0.01). Dex intervention, however, attenuated the apoptosis of CSE‐induced 16HBE cells, downregulated Bax and caspase‐3, and upregulated Bcl‐2 (Figure 2B–E, *p* < 0.05). Meanwhile, the results of ELISA assays showed that CSE markedly elevated the level of IL‐6 (Figure 2F), IL‐8 (Figure 2G), and TNF‐*α* (Figure 2H) in 16HBE cells, all of which were significantly suppressed by Dex treatment (Figure 2F–H, *p* < 0.001).

### 3.3. Effects of miR‐146a on CSE‐Induced Inflammation and Apoptosis of 16HBE Cells

**Figure 3:**
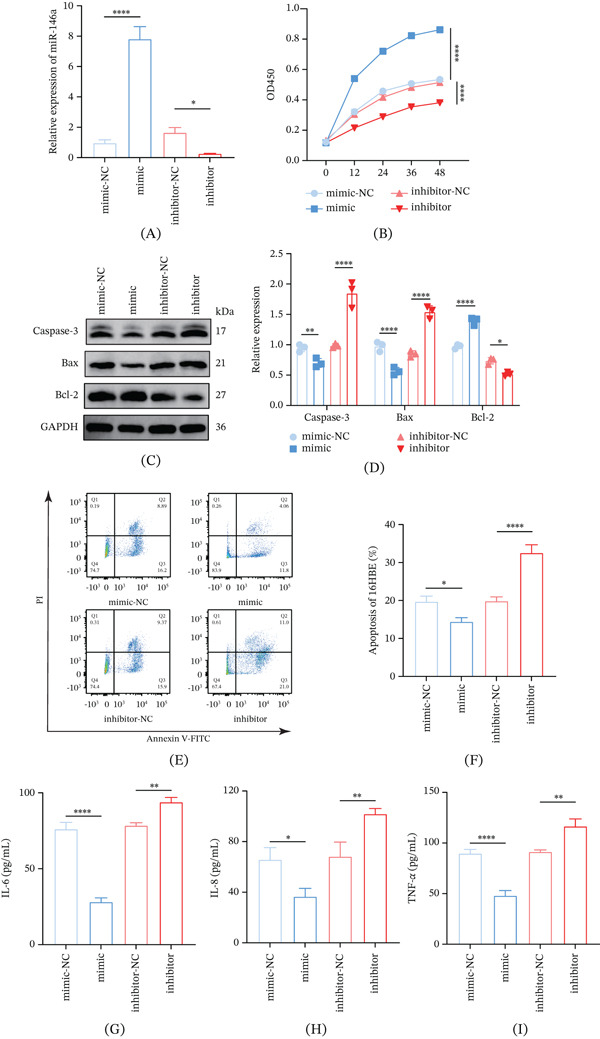
The effect of miR‐146a on CSE‐induced inflammation and apoptosis of 16HBE cells. (A) Expression of miR‐146a quantified via qRT‐PCR. (B) CCK‐8 assay was utilized to measure the effects of miR‐146a on 16HBE viability. (C, D) Relative expression of Bcl‐2, caspase‐3, and Bax in 16HBE cells. (E, F) Flow cytometry determined the apoptosis (%) of 16HBE cells. (G–I) Results of ELISA assay on the concentrations of (G) IL‐6, (H) IL‐8, and (I) TNF‐*α* in 16HBE cells with diverse interventions.  ^∗∗∗∗^ *p* < 0.0001, ^∗∗^ *p* < 0.01, ^∗^ *p* < 0.05.

Successful transfection was confirmed by the significant increase or decrease in the level of miR‐146a in these modeled cells (Figure 3A, *p* < 0.05). Functional assays revealed that in CSE‐induced 16HBE cells, intervention with miR‐146a mimic enhanced the cell viability and Bcl‐2 expression, reduced apoptosis and the levels of Bax and caspase‐3, along with a lower concentration of TNF‐*α*, IL‐6, and IL‐8 (Figure 3 B–I, *p* < 0.05). However, miR‐146a inhibitor produced the opposite effects to the cells across all these parameters (Figure 3B–I, *p* < 0.05).

**Figure 4:**
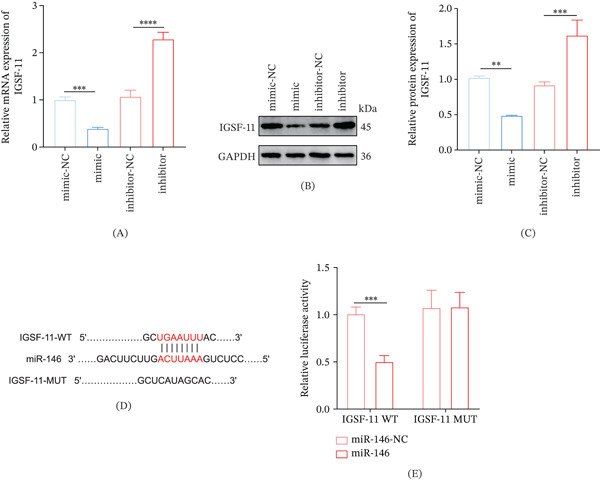
Verification on the targeting correlation between miR‐146a and *IGSF11*. (A) Quantified level of *IGSF11* through qRT‐PCR. (B‐C) Effect of miR‐146a on the protein expression level of *IGSF11* in 16HBE cells via Western blot assay. (D) There are potential binding sites between the miR‐146a and the 3 ^′^ UTR of *IGSF11*. (E) Dual‐luciferase reporter assay validated the targeting correlation between *IGSF11* and miR‐146a. ^∗^ *p* < 0.05,  ^∗∗^ *p* < 0.01,  ^∗∗∗^ *p* < 0.001.

### 3.4. Validation on the Targeting Correlation Between *IGSF11* and miR‐146a

The results showed that *IGSF11* expression was suppressed by miR‐146a mimics but enhanced by miR‐146a inhibitor (Figure 4A–C, *p* < 0.05). Additionally, miR‐146a‐3p and the 3 ^′^ UTR of *IGSF11* shared potential binding sites (Figure 4D). The reduced luciferase activity of *IGSF11*‐WT after the transfection of miR‐146a (Figure 4E, *p* < 0.001) also confirmed the targeting relationship between *IGSF11* and miR‐146a.

### 3.5. Effects of Dex on the Expressions of miR‐146a And *IGSF11* in CSE‐Induced 16HBE Cells

**Figure 5:**
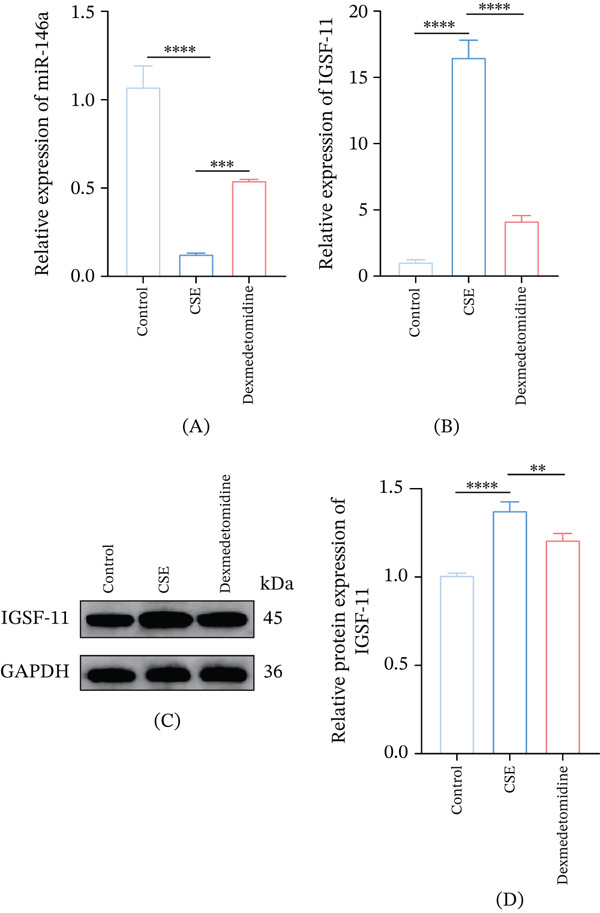
The effect of Dex on the level of miR‐146a and *IGSF11* in CSE‐treated 16HBE cells. (A) Quantified miR‐146a expression level in 16HBE cells with diverse interventions via qRT‐PCR. (B–D) Quantified *IGSF11* expression level with diverse interventions via (B) qRT‐PCR and (C–D) Western blot.  ^∗∗^ *p* < 0.01,  ^∗∗∗^ *p* < 0.001,  ^∗∗∗∗^ *p* < 0.0001.

The effects of Dex on miR‐146a/*IGSF11* axis in CSE‐exposed 16HBE cells were analyzed. We found that CSE suppressed the level of miR‐146a but enhanced that of *IGSF11* in 16HBE cells, whereas Dex treatment downregulated *IGSF11* yet upregulated miR‐146a in CSE‐induced 16HBE cells (Figure 5A–D, *p* < 0.01).

### 3.6. Interplay Between miR‐146a and *IGSF11* on the Inflammation and Apoptosis in CSE‐Induced 16HBE Cells

**Figure 6:**
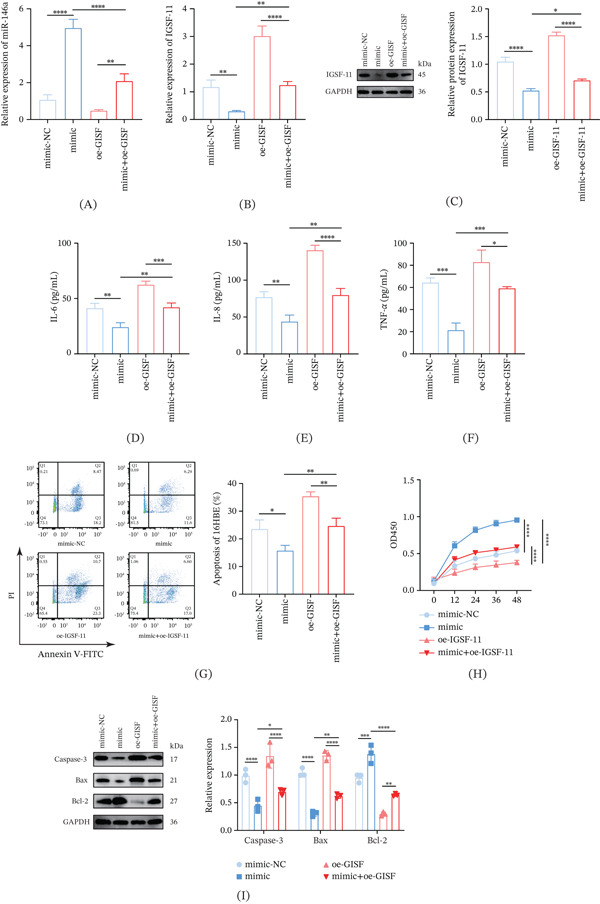
Interplay between miR‐146a and *IGSF11* on the CSE‐induced inflammation and apoptosis of 16HBE cells. (A) Quantified miR‐146a expression level in 16HBE cells with diverse interventions via quantitative real‐time PCR. (B, C) Quantified *IGSF11* expression level with diverse interventions via (B) qRT‐PCR and (C) Western blot. (D–F) Results of ELISA assay on (D) inflammatory cytokines IL‐6, (E) IL‐8, and (F) TNF‐*α* in 16HBE cells with diverse interventions. (G) Apoptosis rate (%) of 16HBE cells determined via flow cytometry. (H) CCK‐8 assay was employed to detect the effects of miR‐146a and *IGSF11* on 16HBE cell viability. (I) Relative expression of Bcl‐2, caspase‐3, and Bax in 16HBE cells.  ^∗^ *p* < 0.05,  ^∗∗^ *p* < 0.01,  ^∗∗∗^ *p* < 0.001, ^∗∗∗∗^ *p* < 0.0001.

The interaction between *IGSF11* and miR‐146a on 16HBE cells exposed to CES was analyzed. Consistent with our previous results, miR‐146a mimic and oe‐*IGSF11* elevated the expression of *IGSF11* and miR‐146a, respectively (Figure 6A–C, *p* < 0.01). Furthermore, in the miR‐146a mimic group, overexpression of *IGSF11* upregulated *IGSF11* and downregulated miR‐146a, respectively (Figure 6 A–C, *p* < 0.01).

ELISA assay (Figure 6D–F), flow cytometry (Figure 6G), CCK‐8 (Figure 6H), and Western blot (Figure 6I) demonstrated that miR‐146a mimic lowered the concentrations of inflammatory cytokines and reduced the apoptosis of CSE‐induced 16HBE cells, along with downregulated caspase‐3 and Bax and upregulated Bcl‐2 (Figure 6D–1, *p* < 0.05). Conversely, overexpressed *IGSF11* produced opposite results and reversed the effects of miR‐146a on the inflammation and apoptosis of CSE‐induced 16HBE cells (Figure 6D–I, *p* < 0.05).

**Figure 7:**
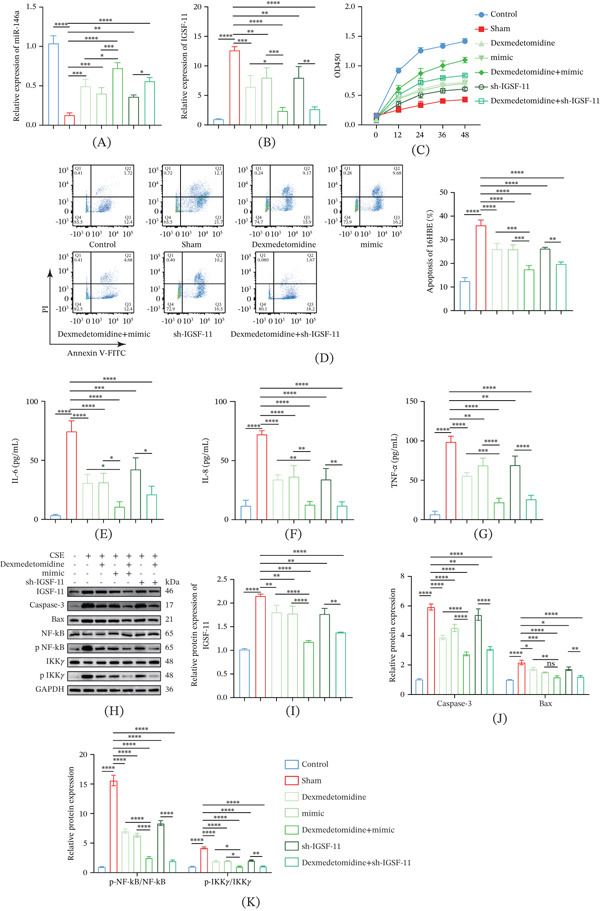
The effect of Dex on the inflammation and apoptosis of CSE‐induced 16HBE cells via targeting miR‐146a/*IGSF11* axis. (A, B) Quantified expression level of (A) miR‐146a and (B) *IGSF11* in each group of 16HBE cells. (C) Viability of 16HBE cells in each group based on CCK‐8 assay. (D) Apoptosis rate (%) of 16HBE cells determined via flow cytometry. (E–G) Results of ELISA assay on the concentrations of (E) IL‐6, (F) IL‐8, and (G) TNF‐*α* in 16HBE cells with diverse interventions. (H–K) Protein expression of *IGSF11* and apoptosis‐ and NF‐*κ*B pathway‐correlated proteins in each group of 16HBE cells, as determined in Western blot assay.  ^∗∗∗∗^ *p* < 0.0001,  ^∗∗∗^ *p* < 0.001, ^∗∗^ *p* < 0.01, ^∗^ *p* < 0.05.

### 3.7. Validation on the Effects of Dex and miR‐146a/*IGSF11* Axis on the Inflammation and Apoptosis of CSE‐Exposed 16HBE Cells

Combined effect of Dex and miR‐146a/*IGSF11* axis on the inflammation and apoptosis of CSE‐exposed 16HBE cells was examined. Consistent with the previous results, CSE upregulated the level of *IGSF11* but downregulated that of miR‐146a, whereas Dex and miR‐146a mimic produced an opposite effect (Figure 7A,B, *p* < 0.05). Also, the knockdown of *IGSF11* and Dex intervention downregulated the level of *IGSF11* (Figure 7A,B, *p* < 0.01).

Additionally, the effects of Dex and miR‐146a/*IGSF11* axis on the inflammation and apoptosis of CSE‐exposed 16HBE cells were determined. CSE suppressed the cell viability and aggravated apoptosis and inflammation, along with upregulated levels of *IGSF11*, inflammatory cytokines, and apoptosis‐associated proteins (caspase‐3 and Bax) (Figure 7C–H, *p* < 0.0001). However, the intervention of Dex with miR‐146a mimic and sh‐*IGSF11* produced opposite results (Figure 7C–G, *p* < 0.05).

Western blot showed that CSE induced the phosphorylation of both NF‐*κ*B and IKK*γ*, and that this activation was suppressed by the treatment of Dex with miR‐146a mimic and sh‐*IGSF11* (Figure 7H–K, *p* < 0.05).

### 3.8. Effects of Dex on the Inflammation of CS‐Induced COPD Rats via Potentially Participating in miR‐146a/*IGSF11*/NF‐*κ*B Axis

**Figure 8:**
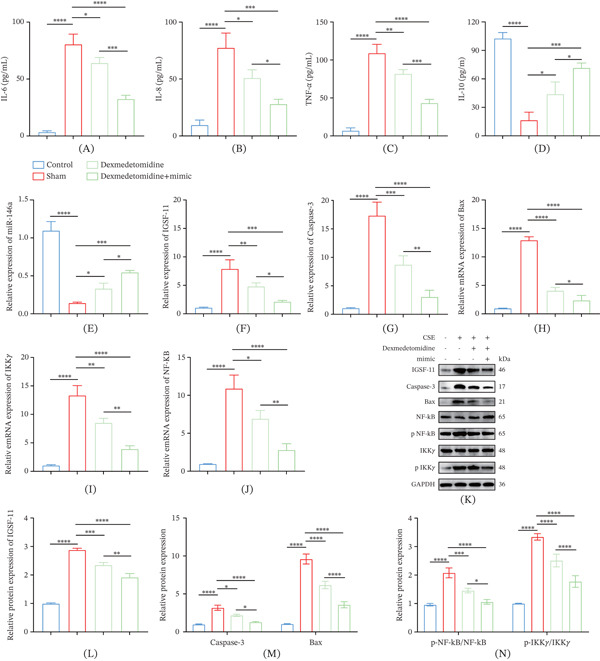
The effect of Dex on the inflammation of CS‐induced COPD rats via targeting miR‐146a/*IGSF11*/NF‐*κ*B axis. (A–D) Results of ELISA assay on the concentrations of (A) IL‐6, (B) IL‐8, (C) TNF‐*α*, and (D) IL‐10 in rats with diverse interventions. (E, F) Quantified expression level of (E) miR‐146a and (F) *IGSF11* in each group of rats based on qRT‐PCR. (G–J) The expressions of apoptosis‐ and NF‐*κ*B pathway‐correlated proteins in each group of rats determined via qRT‐PCR. (K–N) Calculated expression levels of *IGSF11* and apoptosis‐ and NF‐*κ*B pathway‐correlated proteins in each group of rats calculated using Western blot.  ^∗^ *p* < 0.05, ^∗∗^ *p* < 0.01, ^∗∗∗^ *p* < 0.001, ^∗∗∗∗^ *p* < 0.0001.

A CS‐induced COPD rat model was established to verify the mechanism of action of Dex. ELISA assay revealed that CS increased the production of TNF‐*α*, IL‐6, and IL‐8 and reduced the level of anti‐inflammatory cytokine IL‐10 (Figure 8A–D, *p* < 0.0001). However, intervention via Dex and miR‐146a mimic promoted the production of IL‐10 yet suppressed the levels of the three inflammatory cytokines (Figure 8A–D, *p* < 0.01).

Also, the expressions of proteins related to apoptosis and NF‐*κ*B pathway and miR‐146a/*IGSF11* were quantified. It was observed that CS upregulated the expressions of *IGSF11* and proteins associated with apoptosis and the NF‐*κ*B pathway but downregulated that of miR‐146a, whereas both miR‐146a and Dex could weaken such effects of CSE (Figure 8E–N, *p* < 0.05).

## 4. Discussion

COPD is defined by persistent pulmonary inflammation, which leads to progressive and irreversible airflow obstruction [31]. This not only correlates with poor clinical outcomes but also drives frequent acute exacerbation, underscoring the need for effective anti‐inflammatory interventions [32, 33]. Abnormal concentration of inflammatory cells in COPD may lead to abnormal release of cytokines (IL‐6, IL‐8, and TNF‐*α*) [34]. IL‐6 is involved in both innate and adaptive immune response, and excessive response mediated by IL‐6 is detrimental to the host [35, 36]. IL‐8 is a potent neutrophil chemoattractant, with a higher level indicating increased oxidative stress and severity [37, 38]. TNF‐*α*, an inflammatory cytokine, plays a crucial role in inflammation, cytotoxicity, and immune regulation in COPD [39, 40]. In this context, Dex could diminish the levels of these proinflammatory mediators in aged mice with neuroinflammation, as well as in patients undergoing one‐lung ventilation [41, 42]. IL‐10 is a crucial anti‐inflammatory cytokine expressed under excessive regulation of proinflammatory cytokines. Our ELISA assay showed that Dex effectively suppressed the expressions of all these proinflammatory cytokines and promoted the level of IL‐10 [43]. Dex could upregulate the expression of Bcl‐2 and suppress that of Bax and caspase‐3 in rats with diabetic peripheral neuropathy [44]. Consistently, we found that Dex attenuated apoptosis in CSE‐induced 16HBE and CS‐treated rats, along with downregulated Bax and caspase‐3 and upregulated Bcl‐2. These results collectively indicated the antiapoptotic and anti‐inflammatory properties of Dex on COPD.

Previous studies have established that Dex upregulates miR‐146a expression, a mechanism implicated in its protective effects across various injury models [45–47]. For instance, Dex attenuates hypoxia/reoxygenation injury in cardiomyocytes via miR‐146a upregulation through the MAPK signaling pathway [48]. Similarly, in kidney injury, Dex mitigates disease severity while concurrently elevating miR‐146a expression [49]. Most relevant to our study, Dex has been shown to alleviate COPD by promoting miR‐146a expression [12]. Consistently, we found that Dex alleviated COPD progression in CSE‐treated 16HBE cells and CS‐treated rats potentially via the miR‐146a/*IGSF11* axis through upregulating the expression of miR‐146a and downregulating that of *IGSF11*. Given its established role in promoting the invasion and migration of melanoma cells and its association with EMT [23], *IGSF11* was hypothesized to be a key regulator of epithelial barrier integrity and cell adhesion. However, the involvement of *IGSF11* and the interaction between *IGSF11* and miR‐146a in COPD have not been properly examined. This study confirmed the targeting relationship between the two, and further demonstrated that CSE upregulated *IGSF11* expression, which in turn enhanced CSE‐induced apoptosis and inflammation in 16HBE cells. Conversely, silencing *IGSF11* strengthened the protective effects of Dex against these pathological processes. Moreover, the phosphorylation of proteins (NF‐*κ*B and IKK*γ*) correlated to the NF‐*κ*B pathway was enhanced in CSE‐induced 16HBE cells and CS‐treated rats but suppressed by Dex and miR‐146a mimic. The NF‐*κ*B pathway is the central pathway of inflammatory and immune responses. In the canonical NF‐*κ*B pathway, IKK*γ*/NEMO serves as a key regulator for activating NF‐*κ*B [50]. In the noncanonical NF‐*κ*B pathway, IKK*γ* plays an auxiliary role in regulating pathway activation and signal amplification. Collectively, it could be deduced that Dex might protect against inflammation and apoptosis in COPD via potentially participating in the miR‐146a/*IGSF11*/NF‐*κ*B axis.

This study also had certain limitations. First, the sample size was relatively limited, and only in vitro cell experiments and in vivo animal studies were conducted without validation in clinical samples. Second, the animal experiments used exclusively male rats, failing to account for potential impacts of gender differences on the results. This may restrict the generalizability of the present findings. Future research should expand the sample size, incorporate clinical samples from COPD patients for validation, and include female rats in comparative experiments to further consolidate the current findings. Additionally, translational application of this pathway into drug design for COPD and its clinical diagnosis and treatment will require more clinical evidence to support its feasibility, necessitating additional clinical trials.

## 5. Conclusion

Utilizing both CSE‐induced 16HBE cells and a CS‐induced rat model of COPD, this study demonstrated that Dex protected against inflammation and apoptosis in COPD via potentially participating in the miR‐146a/*IGSF11*/NF‐*κ*B axis. These findings validated the effects of Dex and miR‐146a on COPD and provided novel evidence for the prevention of COPD and its treatment.

**Nomenclature**
COPD: chronic obstructive pulmonary disease
Dex: dexmedetomidine
HMGB1: high mobility group box‐1
TLR4: toll‐like receptor 4
NF‐*κ*B: nuclear factor kappa B
ALI: acute lung injury
Treg: regulatory T cells
AMPK: AMP‐activated protein kinase
SIRT1: Sirtuin 1
miRNAs: microRNAs
mRNAs: messenger RNAs
PBMCs: peripheral blood mononuclear cells
CS: cigarette smoke
CSE: cigarette smoke extract
min: minutes
h: hour
IGSF11: immunoglobulin superfamily Member 11
CCK‐8: Cell Counting Kit‐8
FITC: fluorescein isothiocyanate
PI: propidium iodide
PBS: phosphate buffered saline
IL: interleukin
TNF: tumor necrosis factor
IKK*γ*
: I*κ*B kinase gamma
IgCAMs: immunoglobulin superfamily cell adhesion molecules
EMT: epithelial–mesenchymal transition

## Author Contributions

All authors contributed to this present work: X.C., D.W., and N.L. designed the research; J.D., J.L., and Z.W. interpreted the results; B.C., X.Y., S.L., and N.L. analyzed the results; X.C., J.D., Z.W., X.Y., and N.L. drafted the manuscript; D.W., J.L., B.C., S.L., and N.L. revised the manuscript. X.C. and D.W. have equal contributions in this study.

## Funding

This study was supported by the Hainan Province Science and Technology Special Fund (No. ZDYF2024SHFZ117), Hainan Provincial Natural Science Foundation of China (No. 822RC816), and National Natural Science Fund Cultivating 530 Project of Hainan General Hospital (No. 2021MSXM14).

## Disclosure

D.W., J.L., B.C., S.L., and N.L. gave the final approval of the version to be published. All authors read and approved the final manuscript.

## Ethics Statement

The authors have nothing to report.

## Consent

The authors have nothing to report.

## Conflicts of Interest

The authors declare no conflicts of interests.

## Supporting information


**Supporting Information** Additional supporting information can be found online in the Supporting Information section. Table S1: 3 ^′^ UTR sequence of IGSF11.

## Data Availability

The datasets generated and/or analyzed during the current study are available in corresponding authors upon reasonable request.
