# Supplementary material for: The miR‐146a/IGSF11 Axis Potentially Mediates the Protective Effect of Dexmedetomidine Against Cigarette Smoke–Induced Chronic Obstructive Pulmonary Disease
Source: Hum Mutat. 2026 May 13;2026:1858420. doi: 10.1155/humu/1858420 (PMC13171721; doi:10.1155/humu/1858420)
Supplement: Supplementary file 1 — Supporting Information Additional supporting information can be found online in the Supporting Information section. Table S1: 3 ′ UTR sequence of IGSF11. [file HUMU-2026-1858420-s001.docx]

**Supplementary Table 1. 3' UTR sequence of IGSF11**

5’-GACATGAGGAAATGTTGTGTTCAGAAATGAATAAATGGAATGCCCTCATACAAGGGGGAGGGTGGGGTGGGGAGTGCTGGGAAAGAAACACTTCCTTATAATTATATTAGTAAAATGCACAAAGAAGAAGGCAGTGCTGTTACTTGGCCACTAAGATGTGTAAAATGGACTGAAATGCTCCATCATGAAGACTTGCTTCCCCACCAAAGATGTCCTGGGATTCTGCTGGATCTCAAAGATGTGCCAAGCCAAGGAAAAAGATACAAGAGCAGAATAGTACTTAAAATCCAAACTGCCGCCCAGATGGGCTTGTTCTTCATGCCTAACTTAATAATTTTTAAGAGATTAAAGTGCCAGATGGAGTTTAAATATTGAAATTATTTTAAAAGGTAGGTGTCTTTAAGAAAATAACAAGCAACCCTGTGATATGTTCCGTCTCTCCCAATTCCCTCGTTATATAGAGGGCTTAATGGTATAAATGGTTAATATTGGTCCCAACAGGGCTGACTCTTCTATCATATAATCAAAACTTTTTACATGAGCAAAATTCAGTAAGAAATGGGGGAAGACAAAGGAAACGTCTTTGAGAAGCCCCTTCATATTTATTTATTTATCTCTTCCTGAACCATGAATTTCATATGTGGAATATTGCTATATTGACAGATTCTTGCCTGTCTGTGTTATTCTAGGATCTGTTACAGGTCCATGGCAATTACTGTTTATTTTTTCCTGGAAAAATATTTTTTTATAAAAGGCTTTTTTTTTTTTTTAAATACATGAGAGGCATTGGGCTAAGAAAGAAAAGACTGTTGTATAATACCTTGTTCAATGGTTGTATTTAGTGAGCTCATAGAGGTCCATCATATCATGACCGAGCTAGGTTGTGTGGGCAGGAAGGTAGGGCTAAGGGGTTGTAGCCTTGCTGGGCAGCCTCTCAGAGCAAGGTTGTTCAGATCTCCCTTGCTATTACAGTAGGTTACTATTAATGAGGGCAGCACCTGATGCCTTTTGTACTGAGGTATGTAACTTTCTCCTTATTTGACAAGTAGAAGTTAACTTACTTGTCAGGGAGGGCAGACGTTTTTTTGTTCTGTTTCGTTTTTCAAAATAATGCTTTTTGCAAAAGAGGTAAGACTGAGACTAAAGGTGTTATCTTCTGGTGTGCTCCTGGAAGTGTCTACCCTACATTTGTGTCAGCTCAGGGTTGCAGTGTTGCCCAGATGCATTTTACATCACTGTAAAGAGATTACTTTTGTGGTTACTACCTGGCTTGGCTGGCCTTGCGGTTCACCAGATTAATTTACAAACTCCCCCACTTTATTTTGTGCTATGTAGATCTGGCCATACTTGCATTAGTGACTGTCTTGCCTTAACCACACTTAAGCAACCCACAAATTTCTTCTCAGATTTGTTTCCTAGATTACTTATGATACTCATCCCATGTCTCAATAAGAGTGTCTTTTCTTTCTGGATGTGTTCTCTTACTCCCTCTTACCACCATACTTTTTGCTCTCTTCTCCTGCAAGCGTAGTCTTCACAGGGAGTGGCTTCCTGACATTTTTTTCAGTTATGTGAATGAATGGAAACCAACAGCTGCTGCAAACACTGTTTTTCCAAGAAGGCTACACTCAGAACCTAACCATTGCCAACCATTTCAGTATTGATAAAAAGCTGAATTTACTTTAGCATTACTTATTTTTTTTTCCATTTGATGGTTCTTACTTTGTAAAAATTTAAATAAATGAATGTCTATACTTTTTATAAAGAAAAGTGAAAATACCATGACACTGAAAAGATGATGCTATCAGATGCTGTTTAGAAAGCATTTATCTTGCATTTCTTTATTCTTTCTAATTATCTAAAATTCAATAAAATTTTATTCATATAAAATAAGTTGTCATTAATTATCAATACTAACGAGTATGTCATTTTAAAACTTAGTATTCTCTTTAATGTTACAAGA-3’
